# Morphological and Histometric Alterations in Gonadal and Neural Tissues of Native and Non‐Native Fishes From the Lower Doce River Basin in Southeastern Brazil

**DOI:** 10.1111/ahe.70168

**Published:** 2026-08-31

**Authors:** Lucas Marcon, Késsia Leite Souza, Natália Martins Travenzoli, Gustavo Ribeiro Rosa, Mara Luiza de Almeida Santos, Wander Ribeiro Ferreira, Paula Nunes Coelho, Jorge Abdala Dergam dos Santos, Elisabeth Henschel

**Affiliations:** ^1^ Laboratório de Sistemática Molecular e Biologia da Reprodução, Departamento de Biologia Animal Universidade Federal de Viçosa Viçosa Brazil

## Abstract

The ichthyofauna of the lower Doce River basin has been exposed to multiple anthropogenic stressors. Among these, the collapse of the Fundão mining tailings dam in Mariana, Minas Gerais, Brazil, in 2015 resulted in massive fish mortality and widespread contamination of aquatic environments. In this context, morphological and histological analyses of gonadal development and larval malformations are valuable tools for assessing biological responses to environmental degradation. This study was designed as an ecosystem‐level biomonitoring assessment involving native and non‐native species to evaluate gonadal maturation stages (SGM), gonadal histopathology, and craniofacial deformities in fish larvae. Fish were collected from lakes, tributaries, reservoirs, and the main channel of the lower Doce River during rainy and dry seasons using gill nets and cast nets. Histological analyses revealed reproductive alterations, including accumulation of interstitial proteinaceous fluid, oocyte atresia, and yolk liquefaction in several species. Non‐native species exhibited a higher frequency of spawned gonadal maturation stages than native species in the main channel of the Doce River and its tributaries. Morphological and histological analyses of the telencephalon and optic tectum of *Pygocentrus nattereri* and *Prochilodus costatus* larvae revealed tissue eversion and abnormal displacement of neural structures associated with craniofacial deformities. Histometric analyses demonstrated significantly higher nuclear intersection counts and reduced inter‐nuclear distances in normal tissues compared with malformed tissues (*p* < 0.05). The occurrence of reproductive and neural alterations across multiple species suggests ecosystem‐level biological impairment and highlights the value of fish assemblages as indicators of environmental quality in the lower Doce River basin.

## 1 Introduction

The Doce River basin covers an area of approximately 86,000 km^2^ and includes about 230 municipalities. Most of these (86%) are located in the states of Minas Gerais (upper and middle portions) and Espírito Santo (the lower portion of the basin) in Brazil (PIRH 2010). The fish fauna of the Doce River basin has been subjected to a wide range of anthropogenic pressures driven by intense socio‐environmental interests, including the suppression of native vegetation for agriculture, industrial expansion, and the construction of hydroelectric and mining dams (Ferreira et al. 2020; Coelho et al. 2023). The spread of non‐native fish species represents another significant environmental impact that has intensified in recent decades throughout the basin, such as *Cichla* spp., *Oreochromis niloticus* , *Serrasalmus brandtii* , *Parachromis managuensis* , *Plagioscion squamosissimus* and *Pygocentrus nattereri* (Latini et al. 2004; Barros et al. 2012; Assis et al. 2024; Souza et al. 2025).

In recent years, the number of environmental disasters worldwide caused by mining activities has increased, many of which have resulted in long‐term impacts (Carrasco et al. 2005; Bose‐O'Reilly et al. 2018). In Brazil, the collapse of the Fundão dam in the municipality of Mariana (MG), in November 2015, stands out as one of the most significant environmental catastrophes in recent history. This event released over 50 million cubic meters of mining tailings into the Doce River basin, which travelled about 600 km downstream to its mouth in the Atlantic Ocean (Gomes et al. 2017). The tailings were composed mainly of iron (Fe), aluminium (Al), silicon dioxide (SiO_2_), and trace amounts of chromium (Cr), cadmium (Cd), and lead (Pb) (Pires et al. 2003; Escobar 2015; Segura et al. 2016; Gomes et al. 2017; Santana et al. 2026). The immediate environmental impacts included the destruction of riparian vegetation, habitat and microhabitat, and the deaths of tons of fish, leaving the river with critically high levels of turbidity and anoxia. These conditions severely affected the aquatic ecosystem along the entire course of the Doce River, including species of economic and ecological importance (Fernandes et al. 2016).

The stages of gonadal maturation (SGM) in fish are classified based on well‐established macroscopic and histological criteria and play a fundamental role in assessing reproductive stocks, determining maturation frequency, and identifying reproductive periods (dos Santos et al. 2019; Zhukova et al. 2024; Marcon et al. 2025). In this context, morphological analyses have gained prominence as reliable biomarker tools for detecting environmental impacts, particularly through the identification of histopathological alterations in fish organs (Savassi et al. 2016; Marcon et al. 2017). Alterations in gonadal morphology may reflect disruptions in key reproductive processes, including oestrogen regulation (Senthilkumaran 2015), spermatogenesis (Domingos et al. 2012), and folliculogenesis (Chakrabarty et al. 2012; Abdo et al. 2018). Additionally, exposure to environmental stressors can induce histopathological damage in vital organs such as the liver, gills, and spleen, compromising essential physiological functions and overall fish health (Chakrabarty et al. 2012; Marcon et al. 2016; Savassi et al. 2020; Santana et al. 2026).

The early stages of the fish life cycle require special attention, as developmental alterations affect growth and size, sex ratios, and adult behaviour (Jonsson and Jonsson 2014), thereby influencing species' ecological dynamics and persistence. Although early craniofacial deformities play a key role in adult viability, they are rarely reported in the scientific literature (Pereira et al. 2026). Fish embryos and larvae exhibit high sensitivity to environmental stressors, and exposure to pollutants can result in a wide range of malformations, including spinal curvatures, craniofacial deformities, anomalies in the swim bladder, fin, and lateral line system (Usenko et al. 2007; Johnson et al. 2007; Sulukan et al. 2017). Neurological alterations may also occur, including cellular and brain structural damage, loss of synaptic connections, cellular disorganization, necrosis, and ultimately, cell death (Sulukan et al. 2017). These effects may be associated with the induction of reactive oxygen species and subsequent tissue damage (Bagchi et al. 1995). Overall, the effects of pollutants on larval brain structure have been predominantly investigated under controlled laboratory conditions (Sulukan et al. 2017; Horn and Rasia‐Filho 2018), with a notable lack of studies involving wild‐caught fish, particularly the ones from the Doce River basin.

The reproductive and developmental characteristics of species within an ecosystem provide critical insights for conservation strategies and the persistence of viable natural populations (Perrotti et al. 2019; Diniz et al. 2023). Because teleost fishes exhibit considerable diversity in reproductive strategies and developmental patterns, comparative analyses among taxonomic groups often require species‐specific or phylogenetically structured approaches (Melo et al. 2017). However, environmental biomonitoring studies frequently adopt a multispecies framework to evaluate whether anthropogenic stressors produce convergent biological responses across entire fish assemblages (Fausch et al. 1990; van der Oost et al. 2003). Recent studies conducted in the Doce River basin following the Fundão Dam disaster have highlighted the importance of integrating biological responses across multiple species and levels of biological organization to better understand the ecological consequences of large‐scale environmental disturbances (Santana et al. 2026). Under this perspective, the occurrence of similar reproductive and developmental abnormalities across unrelated species may provide important clues for the direction of the fish assemblage under severe ecosystem‐level disruption, particularly in river basins subjected to long‐term contamination and habitat alteration (Fausch et al. 1990; van der Oost et al. 2003). In this context, assessing the reproductive dynamics and neural abnormalities of fish assemblages in the lower Doce River is essential for evaluating environmental quality, as reproductive performance reflects physiological and adaptive responses to the chronic effects of the Fundão Dam collapse. Moreover, these analyses provide an additional tool for assessing short‐ and long‐term effects of the mining disaster on the ichthyofauna.

Therefore, this study aimed to investigate fish reproduction using integrated morphological and histological approaches to quantify the frequency of gonadal maturation stages (SGM) during dry and rainy seasons in native and non‐native fish species from the lower Doce River basin. In addition, we evaluated histopathological alterations in gonadal tissues and morphological abnormalities in the telencephalon and optic tectum of fish larvae to assess reproductive and developmental responses associated with chronic environmental degradation. By integrating reproductive and neural biomarkers across multiple species, this study provides new evidence of sublethal impacts on fish populations inhabiting a historically highly impacted ecosystem.

## 2 Materials and Methods

### 2.1 Animals and Sample Collection

This study is part of the Aquatic Biodiversity Monitoring Program from the Fundação Espírito‐Santense de Tecnologia (PMBA/FEST) following the collapse of mine tailings from the Fundão dam. Adult fish gonad samples were collected monthly during the dry (April to September) and rainy (October to March) seasons, from October 2018 to May 2022, and quarterly from August 2022 to July 2024, using gillnets with mesh sizes ranging from 15 to 70 mm between opposite knots and exposed at 4:00 PM and retrieved at 8:00 PM, providing a total exposition time of 4 h (under IBAMA‐SISBIO licence 64191). The physical and chemical parameters of the water were recorded on the same sections as fish sampling occurred. The following parameters were measured, at 1.5 m depth, with a multi‐parameter instrument Hanna model HI 9829 using pH, temperature (°C), dissolved oxygen (mg/L), and turbidity (NTU) probes. During the study period, the rainy season (October to March) presented mean values of 28.61°C ± 2.19°C for temperature, 5.20 ± 2.20 mg/L for dissolved oxygen, 7.19 ± 0.46 for pH, and 121.01 ± 209.75 NTU for turbidity. The dry season (April to September) presented mean values of 26.31°C ± 2.47°C for temperature, 5.37 ± 2.0 mg/L for dissolved oxygen, 7.21 ± 0.33 for pH, and 13.20 ± 19.02 NTU for turbidity.

**FIGURE 1:**
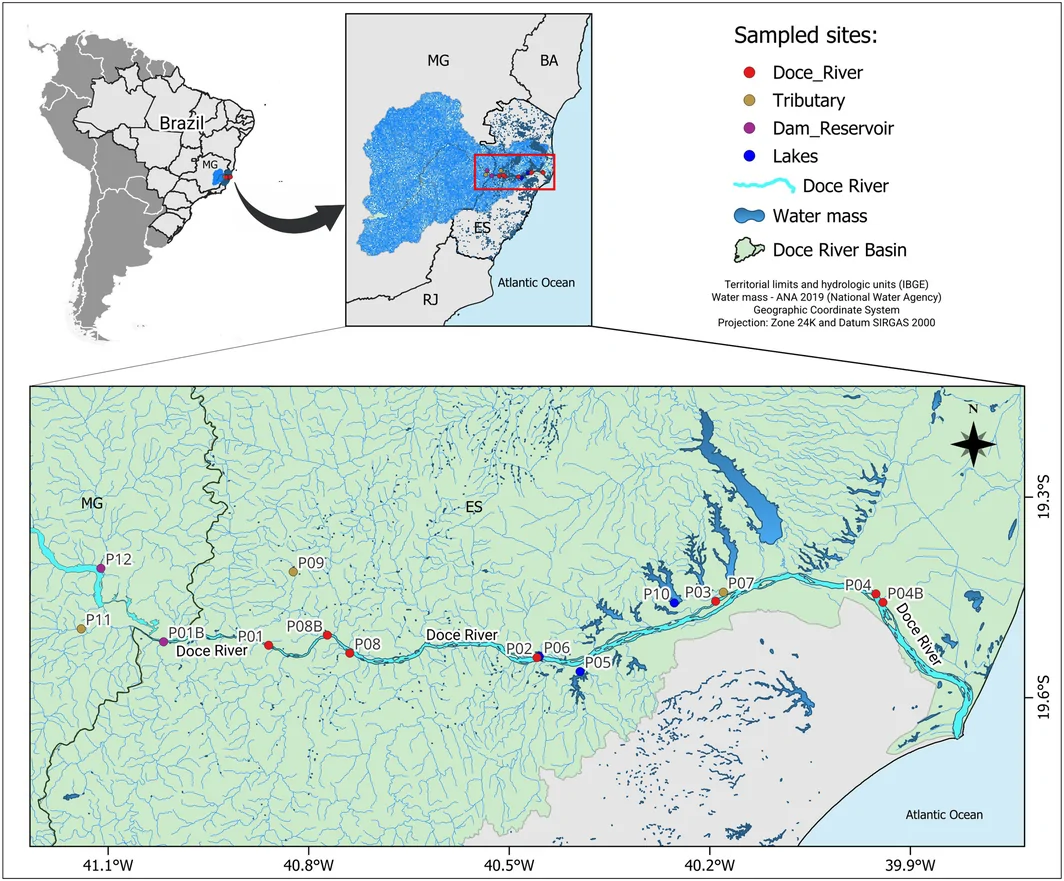
Location of the study area and sampling sites (P01–P12) in the lower Doce River basin, southeastern Brazil. Sampling sites included the main channel of the Doce River, tributaries, lakes, and reservoir.

The sampling locations included the main channel of the Doce River (P1, P2, P3, P4, P8B)—referred to here as Doce River, lakes (P5, P6, and P10), tributaries (P7, P9, and P11), and reservoirs' dam (P01B and P12) in the lower course of the Doce River basin, Espírito Santo state (Figure 1, Table 1). Exceptionally, sites P11 and P12 are located in the state of Minas Gerais and were included in the monitoring program due to the influence of the dam on the downstream section (P12) and the presence of an important tributary—the Manhuaçu River (P11)—which flows between two dams (P12 and P01B). After collection, the fish were anaesthetized with eugenol and fixed in 10% formalin, and were transferred to 70% alcohol. Fish were euthanized according to the standards of the National Council for the Control of Animal Experimentation (CONCEA‐Brazil/2013). Voucher specimens were deposited in the Ichthyology Collection of the João Moojen Zoology Museum, Federal University of Viçosa (MZUFV), Brazil. The research was approved by the Committee on Ethics in the Use of Animals (CEUA of the University Federal of Viçosa, protocol No. 36/2019).

**TABLE 1:** Sampling locations and coordinates of the points in the lower Doce River Basin, in the states of Espírito Santo (ES) and Minas Gerais (MG), UTM coordinates (SIRGAS 2000 UTM Zone 24K).

Site	State	Municipality	Locality	Longitude	Latitude
P01	ES	Baixo Guandu	Doce River	7841208.00	306240.00
P01B	ES	Baixo Guandu	Doce River—Mascarenhas dam reservoir	7841597.00	289743.00
P02	ES	Colatina	Doce River	7839577.00	348378.00
P03	ES	Linhares	Doce River	7849263.00	376384.00
P04	ES	Linhares	Doce River	7850485.00	401503.00
P04B	ES	Linhares	Doce River	7849080.00	402605.00
P05	ES	Colatina	Limão Lake	7837322.00	355148.00
P06	ES	Marilândia	Óleo Lake	7839743.00	348731.00
P07	ES	Linhares	Palmas Stream	7850618.00	377545.00
P08	ES	Colatina	Doce River	7840030.00	318993.00
P08B	ES	Colatina	Doce River	7842984.00	315430.00
P09	ES	Colatina	São Pedro Frio Stream	7853420.00	309975.00
P10	ES	Linhares	Palmas Lake	7848788.00	369855.00
P11	MG	Aimorés	Manhuaçu River	7843587.00	276789.00
P12	MG	Aimorés	Doce River—Aimorés dam reservoir	7853645.00	279749.00

For larval brain region analysis, a conical–cylindrical plankton net (0.5‐mm mesh, 0.12‐m^2^ mouth opening) was used to sample fish larvae. Sampling was conducted at the centre, left, and right banks of the Doce River (P2) for 10 min. The physical integrity of the larvae, including the presence or absence of head deformities, was examined under a stereomicroscope. The neural analyses focused specifically on the telencephalon region and optic tectum region, two major encephalic regions involved in sensory integration, behavioural responses, and environmental perception (Horn and Rasia‐Filho 2018; Perrotti et al. 2019; Pereira et al. 2026). These regions were selected because craniofacial deformities were macroscopically associated with abnormal enlargement and displacement of neural tissues in the dorsal portions of the brain. Only larvae of *Pygocentrus nattereri* (approximately 5 mm total length) and *Prochilodus costatus* (approximately 3 mm total length) were selected for histological and histometric analyses of the telencephalon and optic tectum regions, respectively.

### 2.2 Fixation and Processing for Histology

For histological analyses, fragments from the median region of the gonads of adult fish and whole larvae with normal and abnormal morphology were fixed in 10% formaldehyde for at least 24 h. The samples were dehydrated through a graded ethanol series, embedded in historesin, and sectioned at 3 μm using a rotary microtome (Leica RM2255). Lateral histological sections of the cephalic region larvae (*n* = 5) were stained with toluidine blue (Perrotti et al. 2019; Marcon et al. 2025), DAPI (fluorescence), and colloidal silver solution to identify cell nuclei and argyrophilic nucleolar organizer regions (AgNORs) (Howell and Black 1980).

### 2.3 Histological Analysis and Stages of Gonadal Maturation

The slides were photographed using an Olympus BX53 optical microscope coupled with an Olympus DP73 camera and CellSens Imaging software to highlight potential histopathological changes by comparing the adult gonad fish and larvae with normal and abnormal appearances.

The stages of gonadal maturation (SGM) were quantified and classified using a macroscopic approach, which was subsequently confirmed microscopically for both males (*n* = 2974) and females (*n* = 4441) in native and non‐native species, according to the following criteria: F1/M1 = rest; F2/M2 = initial maturation; F3/M3 = mature; F4/M4 = spawned for females and spent for males (Abdo et al. 2018; dos Santos et al. 2019; Marcon et al. 2025). Subsequently, the relative frequency of SGM in native and non‐native fish was determined for both the rainy and dry seasons throughout the entire sampling period.

### 2.4 Histometric Analysis

For histometric analysis, tissue quantification was performed on normal and abnormal larval specimens of red piranha *Pygocentrus nattereri* and the curimba *Prochilodus costatus* . Images were obtained with a 100× objective and photographed in 10 randomly selected fields. The number of intersection points on neuronal nuclei and the spaces between nuclei were quantified using a grid with 441 intersection points (Marcon et al. 2015; Savassi et al. 2020).

### 2.5 Statistical Analysis

The chi‐squared test (*χ* ^2^; *p* < 0.05) was used to assess the proportion of differences in the quantification of nuclei and spaces between neuronal nuclei in normal and abnormal larvae.

## 3 Results

### 3.1 Histological Analyses and Stages of Gonadal Maturation

**FIGURE 2:**
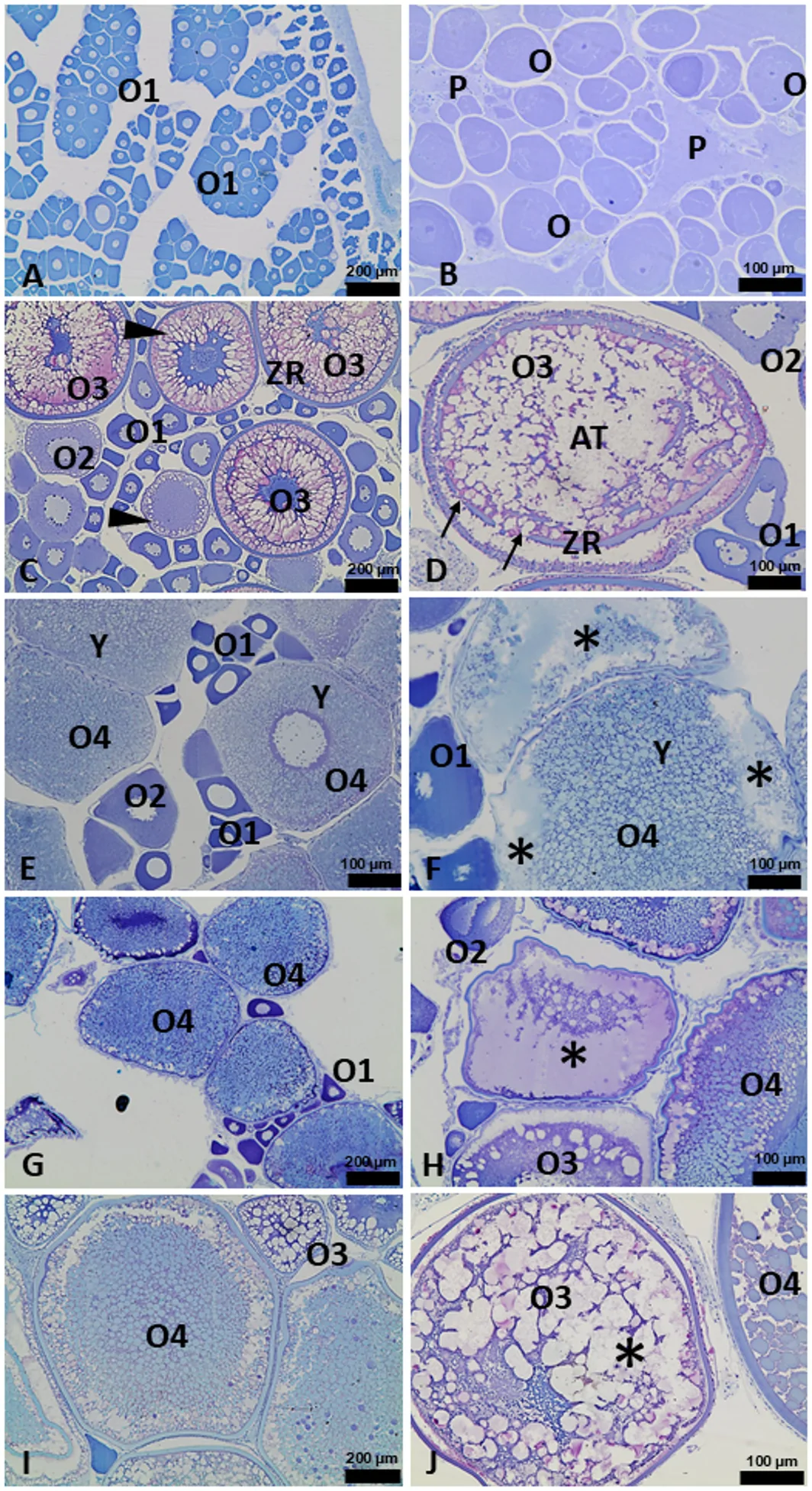
Histological sections of ovaries from fish species collected in the lower Doce River basin stained with Toluidine Blue, showing different stages of gonadal maturation (SGM) and associated histopathological alterations. (A) Rest SGM in *Pimelodus maculatus* showing initial perinucleolar oocytes (O1) characterized by basophilic cytoplasm and advanced perinucleolar oocytes (O2) with enlarged and less basophilic cytoplasm. (B) Detail of the ovarian tissue showing follicular disorganization and accumulation of interstitial proteinaceous material (P) among perinucleolar oocytes (O). (C) Initial maturation SGM in *Metynnis lippincottianus* showing O1, O2, and previtellogenic oocytes (O3) with cortical alveoli in the peripheral ooplasm (arrow head) and zona radiata (ZR) surrounding the oocyte. (D) Detail of an atretic oocyte (AT) with disruption (arrow) of the zona radiata (ZR) in previtellogenic oocytes (O3). (E) Mature SGM in *Astyanax lacustris* showing O1, O2, and vitellogenic oocytes (O4) with cytoplasm densely filled with yolk globules (Y). (F) Exhibits yolk liquefaction (*) accompanied by progressive loss of cytoplasmic homogeneity. (G) Mature SGM in *Knodus moenkhausii* showing O1, O2, O3, and O4. (H) Presents extensive yolk liquefaction (*) within a degenerating vitellogenic oocyte. (I) Mature SGM in *Metynnis lippincottianus* showing O3 and O4. (J) Detail of a yolk liquefaction (*) associated with cytoplasmic disorganization in O3.

Histologically, folliculogenesis was classified into the initial perinucleolar, advanced perinucleolar, previtellogenic, and vitellogenic stages according to the morphological characteristics of the ooplasm and follicular organization (Figure 2A,C,E,G,I). Histopathological alterations were observed in ovaries from several fish species collected throughout the lower Doce River basin. The most common lesions included follicular disorganization and the accumulation of interstitial proteinaceous material among oocytes at different developmental stages (Figure 2B), oocyte atresia associated with disruption of the zona radiata (Figure 2D), and yolk liquefaction accompanied by progressive loss of cytoplasmic homogeneity in vitellogenic oocytes (Figure 2F,H,J).

**FIGURE 3:**
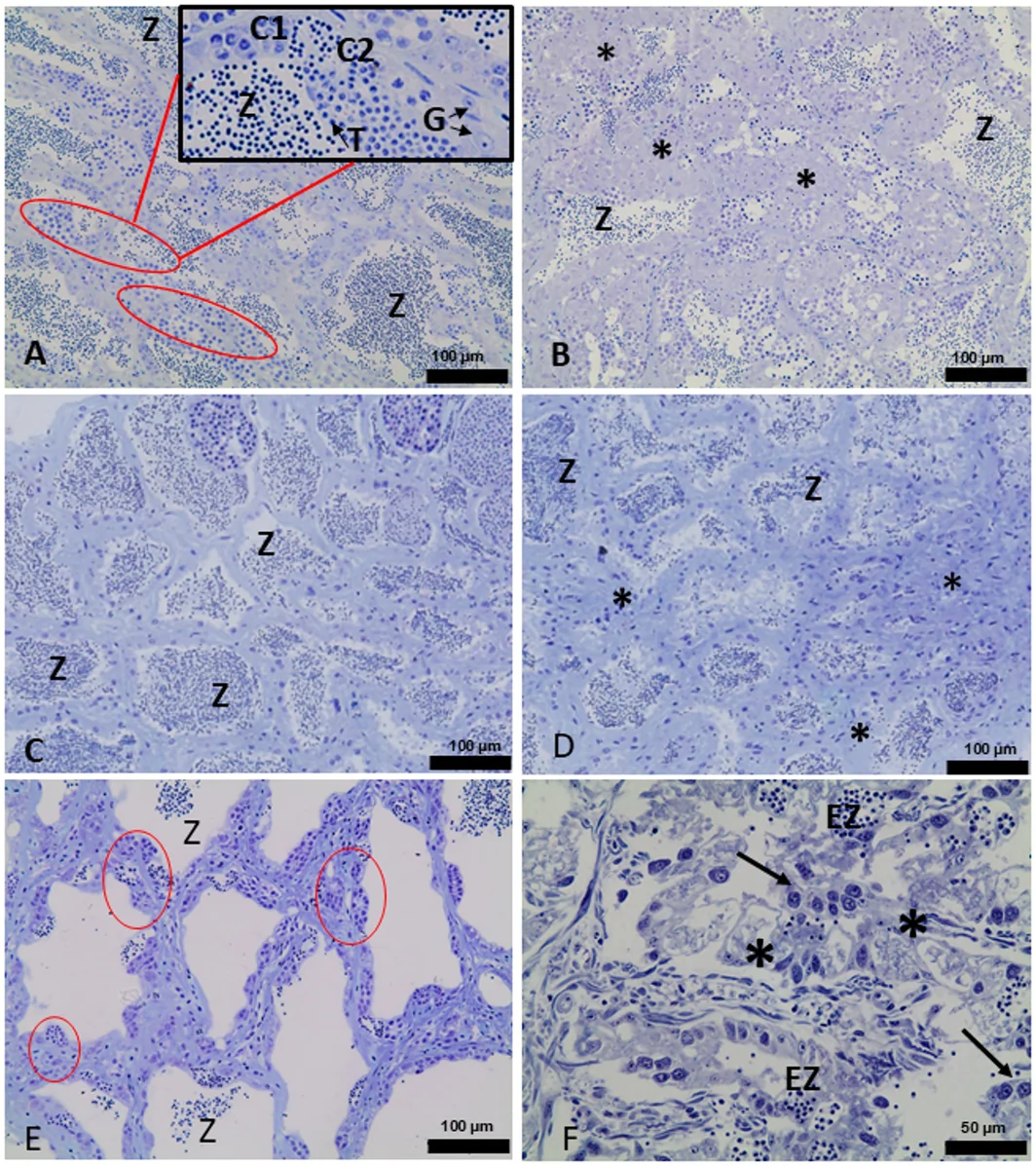
Histological sections of testes from fish collected in the lower Doce River basin, stained with toluidine blue, showing different stages of gonadal maturation (SGM) and associated histopathological alterations. (A) Initial maturation SGM in *Hoplias malabaricus* , showing seminiferous tubules containing spermatozoa (Z) and cysts with different spermatogenic cell stages (red circles). Inset: cells of the spermatogenic lineage, including spermatogonia (G), characterized by abundant cytoplasm and a spherical central nucleus; primary spermatocytes (C1), with a spherical central nucleus and granular chromatin; secondary spermatocytes (C2), with a spherical central nucleus and finely dispersed chromatin; spermatids (T), characterized by a dense spherical nucleus; and spermatozoa (Z), exhibiting a spherical head and a strongly condensed nucleus. (B) Detail of the altered testicular tissue in *H. malabaricus* showing increased interstitial connective tissue thickness (*) and altered testicular architecture. (C) Mature SGM in *Hyporhamphus roberti* , characterized by seminiferous tubules densely filled with spermatozoa (Z). (D) Detail of the altered testicular tissue in *H. roberti* showing increased interstitial connective tissue thickness (*) and altered testicular architecture. (E) Spent SGM in *Hypostomus affinis* , showing seminiferous tubules with little or absent spermatozoa (Z) and cysts containing different spermatogenic cell stages (red circles). (F) Detail of the altered testicular tissue in *H. affinis* showing spermatozoa dispersed outside of the seminiferous tubule lumen (EZ), hypertrophied cells (arrows), inflammatory cell infiltration, and increased connective tissue thickness (*).

During spermatogenesis, all stages of the spermatogenic lineage were observed, including spermatogonia, primary spermatocytes, secondary spermatocytes, and spermatids organized within cysts, as well as seminiferous tubules containing spermatozoa (Figure 3A, inset, C and E). Histopathological alterations were identified in the testes of several species and were characterized by increased interstitial connective tissue thickness and altered testicular architecture (Figure 3B,D), as well as hypertrophied cells and inflammatory cell infiltration (Figure 3F). In addition, spermatozoa were observed dispersed outside the seminiferous tubule lumen, indicating disruption of normal testicular organization (Figure 3F).

**FIGURE 4:**
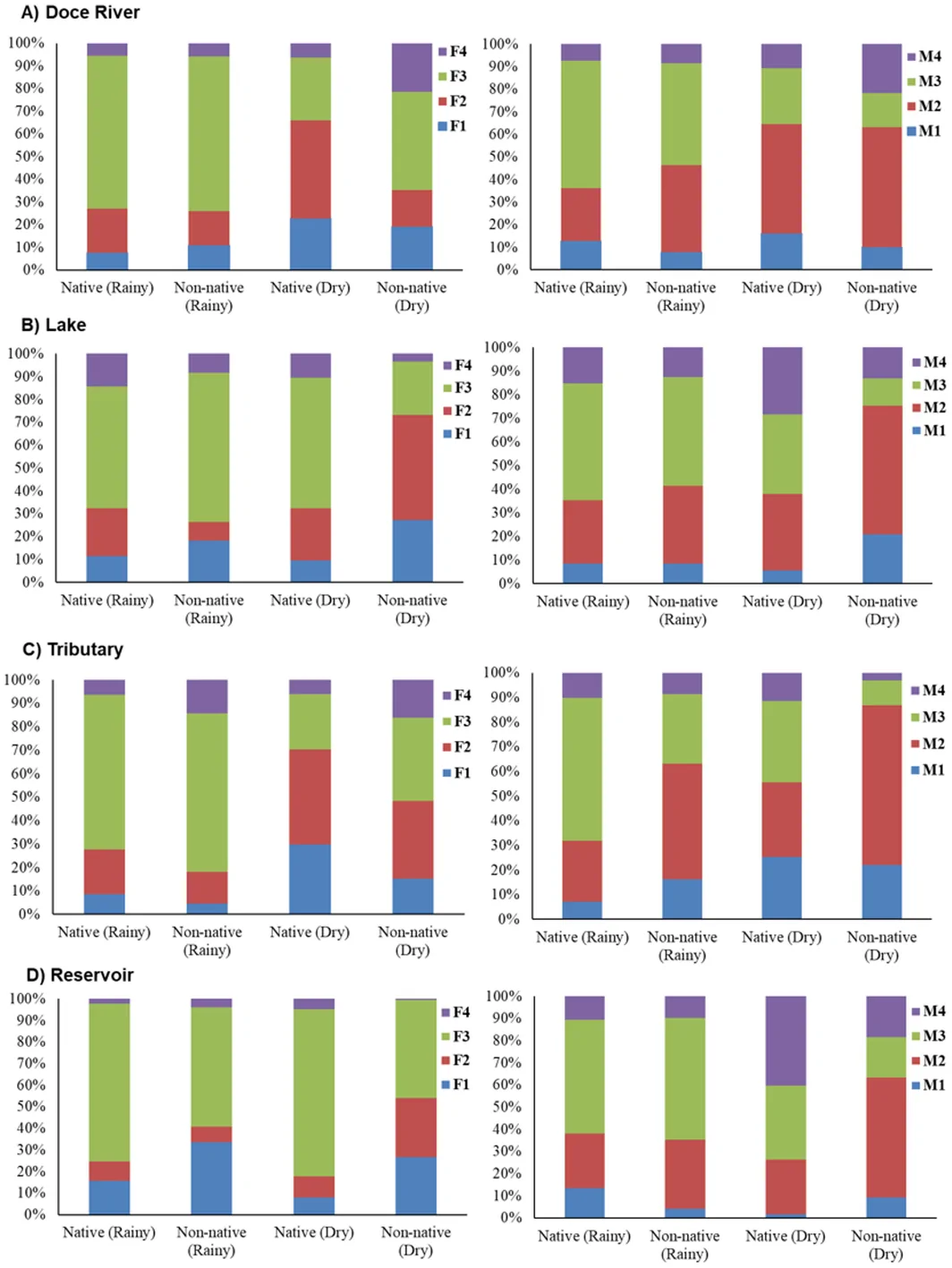
Relative frequency of gonadal maturation stages (GMS) in native and non‐native species of females and males in the Doce River channel (A), Lake (B), Tributary (C), and Reservoir (D) during campaigns conducted from October 2018 to July 2024. Membranous covering surrounding the dorsal telencephalon.

The morphological characteristics of the stages of gonadal maturation (SGM) were characterized in 7415 specimens and identified in 59 fish species, including 38 native and 21 non‐native species (Table 2). The relative frequency of SGM in rest (F1) and initial maturation (F2) predominated in both native and non‐native females during the dry season across all studied environments, as well as in males during rest (M1) and initial maturation (M2), when compared to the rainy season (Figure 4A–D). The stages of gonadal maturation (SGM) in mature (F3/M3) and spawned/spent (F4/M4) fish were recorded with higher relative frequency in both native and non‐native species during the rainy season in all four environments analysed (Figure 4A–D). In the Doce River and tributary, females of non‐native species exhibited a higher relative frequency of the spawned (F4) SGM compared to native species, while males with spent (M4) SGM showed the same pattern in the Doce River.

**TABLE 2:** List of species collected from October 2018 to July 2024, their historical origin in the basin, number of males and females analysed, and mean of total length.

Order | Family | Species	Origin	Mean of total length (TL)	
		Females	Males
**Acanthuriformes**			
Gerreidae			
*Eucinostomus argenteus* ^a^	Native	*n* = 0	*n* = 1 (10.5 cm)
*Eugerres brasilianus* ^a^	Native	*n* = 1 (29.40 cm)	*n* = 1 (29.08 cm)
Sciaenidae			
*Pachyurus adspersus*	Native	*n* = 150 (19.53 cm)	*n* = 145 (17.02 cm)
**Beloniformes**			
Hemiramphidae			
*Hyporhamphus roberti* ^a^	Native	*n* = 4 (12.00 cm)	*n* = 15 (10.30 cm)
**Carangiformes**			
Carangidae			
*Caranx latus* ^a^	Native	*n* = 0	*n* = 1 (20.64 cm)
Centropomidae			
*Centropomus parallelus* ^a^	Native	*n* = 13 (29.90 cm)	*n* = 15 (24.44)
**Characiformes**			
Anostomidae			
*Megaleporinus conirostris*	Native	*n* = 4 (26.97 cm)	*n* = 25 (28.00 cm)
Bryconidae			
*Salminus brasiliensis*	Non‐native	*n* = 3 (36.44 cm)	*n* = 1 (46.17 cm)
Acestrorhamphidae			
*Astyanax lacustris*	Native	*n* = 498 (9.24 cm)	*n* = 465 (8.14 cm)
*Deuterodon* cf. *giton*	Native	*n* = 134 (5.31 cm)	*n* = 50 (4.91 cm)
*Deuterodon* cf. *intermedius*	Native	*n* = 354 (5.45 cm)	*n* = 157 (5.03 cm)
*Deuterodon* cf. *taeniatus*	Native	*n* = 70 (5.76 cm)	*n* = 58 (5.46 cm)
*Megalamphodus eques*	Non‐native	*n* = 196 (3.26 cm)	*n* = 20 (3.24 cm)
*Moenkhausia vittata*	Native	*n* = 31 (5.52 cm)	*n* = 2 (6.02 cm)
*Oligosarcus acutirostris*	Native	*n* = 47 (15.41 cm)	*n* = 22 (13.92 cm)
*Oligosarcus argenteus*	Native	*n* = 3 (13.48 cm)	*n* = 5 (13.33 cm)
*Psalidodon* aff. *fasciatus*	Native	*n* = 22 (5.34 cm)	*n* = 12 (5.32 cm)
Characidae			
*Serrapinnus heterodon*	Native	*n* = 40 (4.06 cm)	*n* = 14 (4.15 cm)
Crenuchidae			
*Characidium cricarense*	Native	*n* = 14 (5.07 cm)	*n* = 5 (7.18)
Erythrinidae			
*Hoplerythrinus unitaeniatus*	Native	*n* = 6 (12.10 cm)	*n* = 8 (20.88 cm)
*Hoplias intermedius*	Native	*n* = 10 (36.20 cm)	*n* = 14 (25.41 cm)
*Hoplias* aff. *malabaricus*	Native	*n* = 79 (24.91 cm)	*n* = 72 (25.88 cm)
Prochilodontidae			
*Prochilodus argenteus*	Non‐native	*n* = 6 (46.51 cm)	*n* = 10 (48.10 cm)
*Prochilodus costatus*	Non‐native	*n* = 26 (34.84 cm)	*n* = 13 (36.99 cm)
Serrasalmidae			
*Metynnis lippincottianus*	Non‐native	*n* = 413 (13.59 cm)	*n* = 299 (12.41 cm)
*Pygocentrus nattereri*	Non‐native	*n* = 193 (20.68 cm)	*n* = 172 (19.89 cm)
*Serrasalmus brandtii*	Non‐native	*n* = 23 (15.89 cm)	*n* = 25 (15.97 cm)
Stevardiidae			
*Knodus moenkhausii*	Non‐native	*n* = 541 (4.05 cm)	*n* = 194 (3.83 cm)
**Cichliformes**			
Cichlidae			
*Aequidens* cf. *plagiozonatus*	Non‐native	*n* = 41 (12.51 cm)	*n* = 33 (11.76 cm)
*Astronotus crassipinnis*	Non‐native	*n* = 1 (21.81 cm)	*n* = 1 (19.74 cm)
*Astronotus ocellatus*	Non‐native	*n* = 0	*n* = 1 (17.39 cm)
*Cichla kelberi*	Non‐native	*n* = 16 (28.13 cm)	*n* = 31 (29.93 cm)
*Cichla monoculus*	Non‐native	*n* = 30 (22.87 cm)	*n* = 47 (21.66 cm)
*Coptodon rendalli*	Non‐native	*n* = 2 (23.53 cm)	*n* = 3 (17.68 cm)
*Saxatilia lepidota*	Non‐native	*n* = 43 (13.41 cm)	*n* = 56 (16.48 cm)
*Geophagus* aff. *brasiliensis*	Native	*n* = 191 (14.50 cm)	*n* = 123 (15.47 cm)
*Oreochromis niloticus*	Non‐native	*n* = 37 (11.83 cm)	*n* = 26 (11.15 cm)
**Clupeiformes**			
Engraulidae			
*Anchoviella cayennensis* ^a^	Native	*n* = 1 (16.79 cm)	*n* = 0
*Lycengraulis grossidens* ^a^	Native	*n* = 61 (9.95 cm)	*n* = 39 (9.78 cm)
**Elopiformes**			
Elopidae			
*Elops saurus* ^a^	Native	*n* = 0	*n* = 1 (44.19 cm)
**Gobiiformes**			
Oxudercidae			
*Awaous tajasica* ^a^	Native	*n* = 460 (6.91 cm)	*n* = 244 (7.90 cm)
**Gymnotiformes**			
Gymnotidae			
*Gymnotus sylvius*	Non‐native	*n* = 9 (28.48 cm)	*n* = 7 (31.67 cm)
**Mugiliformes**			
Mugilidae			
*Mugil curema* ^a^	Native	*n* = 6 (31.17 cm)	*n* = 19 (24.05 cm)
**Siluriformes**			
Ariidae			
*Genidens genidens* ^a^	Native	*n* = 24 (28.52 cm)	*n* = 5 (28.26 cm)
Auchenipteridae			
*Pseudauchenipterus affinis*	Native	*n* = 19 (12.04 cm)	*n* = 10 (10.92 cm)
*Trachelyopterus striatulus*	Native	*n* = 60 (14.78)	*n* = 66 (15.76)
Callichthyidae			
*Osteogaster aenea*	Native	*n* = 1 (6.61 cm)	*n* = 1 (4.91 cm)
*Hoplosternum littorale*	Non‐native	*n* = 188 (14.84 cm)	*n* = 121 (18.15 cm)
Clariidae			
*Clarias gariepinus*	Non‐native	*n* = 2 (53.20 cm)	*n* = 2 (69.10 cm)
Heptapteridae			
*Pimelodella lateristriga*	Native	*n* = 11 (7.81 cm)	*n* = 7 (8.49 cm)
*Rhamdia quelen*	Native	*n* = 6 (16.87 cm)	*n* = 7 (14.81 cm)
Loricariidae			
*Hypostomus affinis*	Native	*n* = 13 (27.26 cm)	*n* = 18 (28.78 cm)
*Hypostomus luetkeni*	Native	*n* = 2 (22.81 cm)	*n* = 1 (18.28 cm)
*Loricariichthys castaneus*	Native	*n* = 58 (30.18 cm)	*n* = 34 (29.36 cm)
*Pterygoplichthys pardalis*	Non‐native	*n* = 13 (32.79 cm)	*n* = 9 (46.01)
Pimelodidae			
*Pimelodus maculatus*	Non‐native	*n* = 237 (23.46 cm)	*n* = 226 (20.94 cm)
Pseudopimelodidae			
*Lophiosilurus alexandri*	Non‐native	*n* = 3 (48.16 cm)	*n* = 0
Trichomycteridae			
*Trichomycterus immaculatus*	Native	*n* = 14 (9.42 cm)	*n* = 15 (8.48 cm)
**Synbranchiformes**			
**Syngnathiformes**			
Syngnathidae			
*Microphis lineatus* ^a^	Native	*n* = 5 (12.24 cm)	*n* = 0
Total number of individuals analysed		*n* = 7415	

^a^ Species from marine, estuarine habitats and mean of total length (TL).

### 3.2 Histological Analyses of Larval Neural Tissue

**FIGURE 5:**
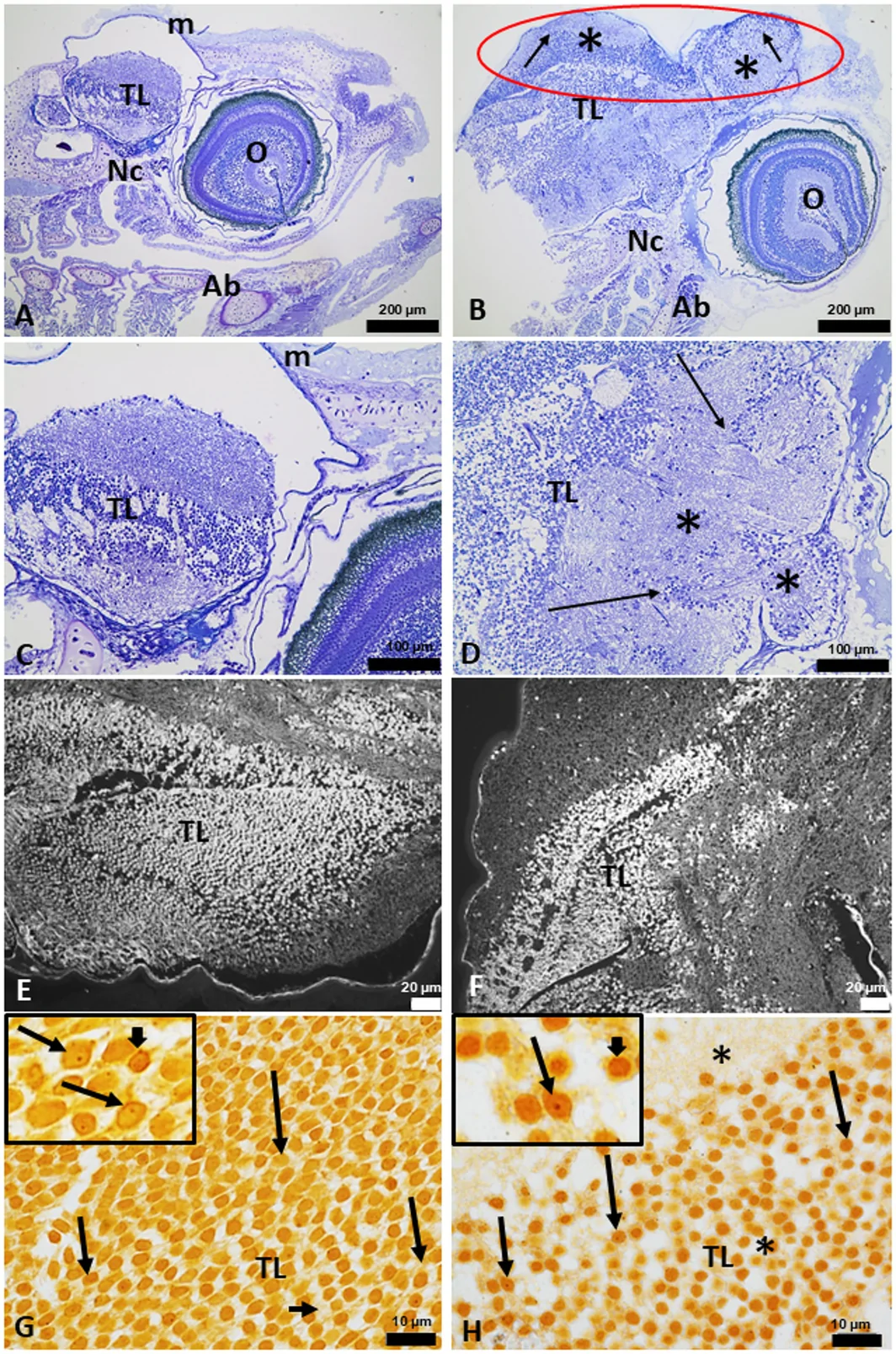
Morphological and histological alterations in the telencephalon region of *Pygocentrus nattereri* larvae stained with toluidine blue, colloidal silver solution, and labelled with DAPI fluorescence. (A) Normal larva showing the telencephalon region (TL) within the normal cranial boundaries, delimited by a thin membranous covering (m) and the developing neurocranium (Nc). (B) Craniofacial deformity characterized by abnormal enlargement of the encephalic region and protrusion of neural tissue beyond the normal cranial boundaries (arrows and red circle), associated with tissue eversion (*). (C) Higher magnification of the normal telencephalon region (TL) showing compact and organized neural tissue enclosed by the membranous covering (m). (D) Higher magnification of the altered telencephalon region showing reduced cellular compactness, displacement of neuronal cells (arrows), and disruption of the normal neural architecture (*). (E, F) DAPI fluorescence labelling showing the distribution of cell nuclei in normal (E) and malformed (F) larvae. (G) Higher magnification of the normal telencephalon region stained with colloidal silver solution showing neuronal cells (arrows). (H) Higher magnification of the altered telencephalon region showing a dispersed distribution of neuronal cells (arrows) and altered neural tissue organization (*). Insets: higher magnification of the neuronal cells with euchromatic nuclei and a prominent central nucleolus (arrow), and glial cells (arrowhead) with heterochromatic nuclei. Ab, branchial arch; m, membranous covering; Nc, developing neurocranium; O, eye; TL, telencephalon region.

**FIGURE 6:**
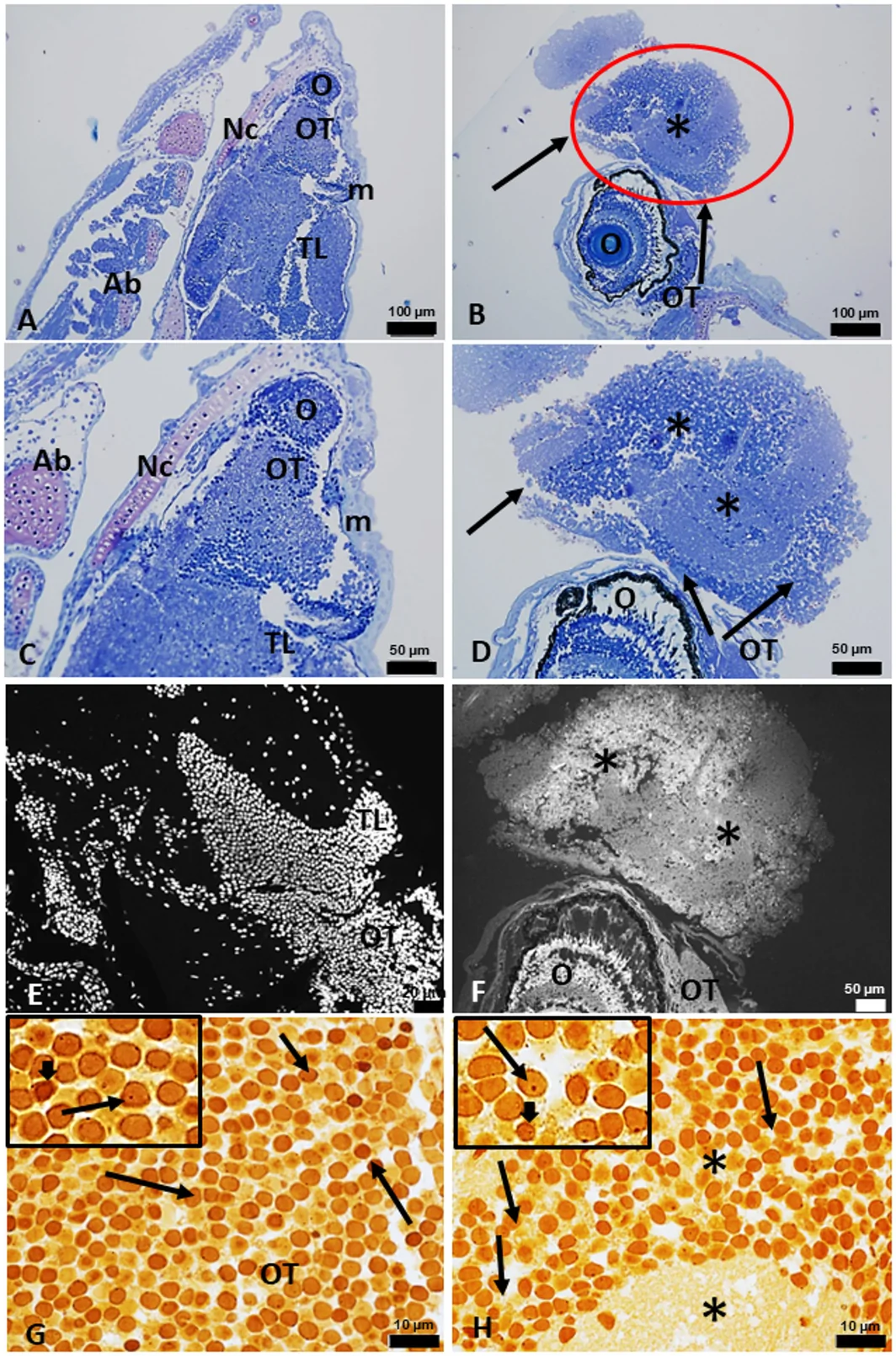
Morphological and histological alterations in the optic tectum region of *Prochilodus costatus* larvae stained with toluidine blue, colloidal silver solution, and labelled with DAPI fluorescence. (A) Normal larva showing the optic tectum region (OT) within the normal cranial boundaries, delimited by a thin membranous covering (m) and the developing neurocranium (Nc). (B) Cranial deformity characterized by abnormal enlargement of the encephalic region and protrusion of neural tissue beyond the normal cranial boundaries (arrows and red circle), associated with neural tissue eversion (*) adjacent to the optic tectum region. (C) Higher magnification of the normal optic tectum region (OT) showing compact and organized neural tissue enclosed by the membranous covering (m). (D) Higher magnification of the altered optic tectum region showing reduced cellular compactness, displacement of neuronal cells (arrows), and disruption of the normal neural architecture (*). (E, F) DAPI fluorescence labelling showing the distribution of cell nuclei in normal (E) and malformed (F) larvae. In malformed larvae, neural tissue eversion (*) was observed adjacent to the optic tectum region (OT). (G) Higher magnification of the normal optic tectum region stained with colloidal silver solution showing neuronal cells (arrows). (H) Higher magnification of the altered optic tectum region showing a dispersed distribution of neuronal cells (arrows) and altered neural tissue organization (*). Insets: higher magnification of neuronal cells with euchromatic nuclei and a prominent central nucleolus (arrows), and glial cells (arrowhead) with heterochromatic nuclei. Ab, branchial arch; m, membranous covering; Nc, developing neurocranium; O, eye; OT, optic tectum region; TL, telencephalon region.

Morphological analyses of the telencephalon region in *Pygocentrus nattereri* and the optic tectum region in *Prochilodus costatus* revealed marked differences between normal and malformed larvae. In normal larvae, both encephalic regions were contained within the normal cranial boundaries and exhibited typical morphology (Figures 5A and 6A). In contrast, malformed larvae exhibited abnormal enlargement of these encephalic regions, characterized by protrusion and eversion of neural tissue beyond the normal cranial boundaries (Figures 5B and 6B). Histological analyses demonstrated that normal larvae exhibited compact and organized neural tissue with densely packed neuronal cells (Figures 5C and 6C). In malformed larvae, the telencephalon region and the neural tissue associated with the optic tectum region showed reduced cellular compactness, displacement of neuronal cells, and disruption of the normal neural architecture (Figures 5D and 6D).

Histological analyses of the telencephalon region and the optic tectum region revealed DAPI‐labelled nuclei (Figures 5E,F and 6E,F). In normal larvae of *P. nattereri* and *P. costatus* , neurons exhibited euchromatic nuclei with a prominent central nucleolus, which were clearly visualized in sections stained with colloidal silver solution (Figures 5G and 6G, insets). In addition, glial cells with heterochromatic nuclei were observed throughout the nervous tissue (Figures 5G and 6G, insets). In larvae exhibiting morphological alterations, the same cellular types were identified in the telencephalon region and the optic tectum region; however, they displayed a more dispersed distribution and reduced cellular organization compared with normal larvae (Figures 5H and 6H, insets).

Histometric analyses demonstrated a higher number of intersection points on neuronal nuclei and reduced spacing between neuronal nuclei in normal larvae compared to abnormal larvae (*p* < 0.05) for both species. In *Pygocentrus nattereri* , chi‐squared (*χ* ^2^) values were 19.513 (*p* < 0.05) for intersection points on neuronal nuclei and 9.856 (*p* < 0.05) for spaces between nuclei. In *Prochilodus costatus* , chi‐squared values of 18.660 (*p* < 0.05) for neuronal nuclei and 5.686 (*p* < 0.05) for spaces between nuclei were recorded.

## 4 Discussion

The presence of histopathological alterations in the gonads, such as yolk globule liquefaction in oocytes and disorganization of the seminiferous tubules, recorded in native species (*Astyanax lacustris*, *Hyporhamphus roberti* , *Hoplias malabaricus* and *Hypostomus affinis* ), as well as in non‐native species (*Knodus moenkhausii*, *Metynnis lippincottianus* and *Pimelodus maculatus* ), provides robust evidence of reproductive impairment associated with environmental degradation. These histopathological characteristics are similar to those reported in other studies of environments contaminated by heavy metals (Savassi et al. 2016, 2020). Specifically, Merçon et al. (2021) observed similar alterations in *Astyanax lacustris* from the lower Doce River, where aluminium (Al) and iron (Fe) were also among the most abundant metals in the water, with concentrations of 543.78 ± 125.58 μg/L^1^ and 491.59 ± 97.36 μg/L^1^, respectively. Research by De Morais et al. (2025) in an aquarium system using water collected from the upper Doce River after the dam rupture also showed negative impacts on *Astyanax lacustris* reproduction, including a higher frequency of atresia and a reduction in oocyte diameter. Furthermore, studies in both the upper and lower Doce River basins have identified the accumulation of arsenic, mercury, and other heavy metals in the aquatic biota across freshwater, coastal, and marine ecosystems (Ferreira et al. 2020; Costa et al. 2022; De Carvalho et al. 2025).

The detection of these lesions across taxonomically and ecologically distinct groups further supports the presence of a widespread, non‐species‐specific stressor affecting the aquatic ecosystem. Therefore, it is plausible that contamination by metals and pesticides in the Doce River basin has contributed to medium‐ and long‐term cellular tissue alterations in the analysed species. Such changes may impair gonadal development and the functional integrity of reproductive processes in fish from the lower Doce River. Additionally, these environmental pressures may constitute one of the underlying factors contributing to the increasing records of non‐native species in the lower Doce River (Souza et al. 2025), potentially reflecting ecological imbalances that favour opportunistic or pollution‐tolerant species over native assemblages. This multispecies pattern reinforces the usefulness of reproductive biomarkers as indicators of ecosystem‐level environmental degradation (van der Oost et al. 2003). Furthermore, despite the well‐recognized diversity of reproductive and developmental strategies among teleost fishes (Melo et al. 2017; Marcon et al. 2025), the recurrent occurrence of similar gonadal and neural alterations across multiple taxa provides additional evidence that these abnormalities may reflect ecosystem‐wide biological responses to chronic environmental degradation rather than isolated taxon‐specific effects. Therefore, the multispecies approach adopted in this study provides a broader assessment of environmental quality in the lower Doce River basin and complements species‐specific investigations by identifying convergent reproductive and developmental biomarkers of ecological disturbance across fish assemblages (Fausch et al. 1990; van der Oost et al. 2003).

The stages of gonadal maturation (SGM) in native and non‐native fish species in the lower Doce River exhibited morphological changes throughout gonadal development, including cellular and structural differentiation. These findings are consistent with observations in diverse groups of teleost fish (Melo et al. 2017; dos Santos et al. 2019; Zhukova et al. 2024; Marcon et al. 2025; Da Silva et al. 2026). The frequency of mature (F3/M3), spawned (F4), and spent (M4) SGM stages in different environments of the lower Doce River (Doce River, lake, tributary, and reservoir) was higher during the rainy periods, aligning with favourable abiotic factors such as temperature, rainfall, and suitable physicochemical conditions for fish growth and reproduction in the Neotropical region (Diniz et al. 2023; Marcon et al. 2025; Da Silva et al. 2026). These favourable environmental conditions stimulate the hypothalamic–pituitary axis to release gonadotropins (GnRH) (Arantes et al. 2010), which, in turn, triggers the initial stages of maturation and reproduction in fish. Overall, non‐native fish are known for their high adaptability and their direct role in reducing native species populations (Beatty et al. 2013; Fragoso‐Moura et al. 2016; Pelicice et al. 2017; Souza et al. 2025). In the present study, a higher relative frequency of mature (F3), spawned (F4), and spent (M4) SGM was observed in non‐native species in both the Doce River and tributaries, raising significant concerns about the potential threat to native species populations in the lower Doce River.

Morphological and histological evaluations of the telencephalon region and the optic tectum region of *Pygocentrus nattereri* and *Prochilodus costatus* revealed that normal larvae exhibited a well‐organized neural architecture composed of euchromatic neurons with prominent nucleoli and glial cells distributed throughout the nervous tissue. In contrast, malformed larvae presented displacement of neural tissue, altered spatial organization of neuronal and glial cells, and tissue eversion affecting both encephalic regions. In teleost larvae, normal brain development is characterized by the progressive organization and compaction of neuronal and glial cells, which establish the structural framework necessary for neuronal differentiation, synaptogenesis, and the formation of functional neural circuits (Horn and Rasia‐Filho 2018; Perrotti et al. 2019). During normal development, this process is reflected by the orderly distribution of neural cells and the preservation of tissue integrity within developing encephalic regions (Perrotti et al. 2019; Horn and Rasia‐Filho 2018). In contrast, disturbances in neural tissue organization, including reduced cellular compactness, abnormal cell displacement, and tissue eversion, may indicate disruptions in neurodevelopmental processes such as cell proliferation, migration, and differentiation. Similar alterations have been associated with impaired neural connectivity, abnormal brain development, and reduced functional integration of neural regions in fish exposed to environmental contaminants and other stressors (Usenko et al. 2007; Sulukan et al. 2017).

Histopathological alterations indicative of neural impairment were detected in both larval species, suggesting that the observed structural abnormalities may compromise neuronal connectivity and synaptic transmission, ultimately affecting central nervous system function (De Bruin 1980; Horn and Rasia‐Filho 2018). According to Bagchi et al. (1995) and Sulukan et al. (2017), brain malformations may result from contaminant‐induced apoptosis and oxidative stress, which can trigger cellular damage and impair normal neural development. Furthermore, a relationship may exist between these mechanisms and the tissue alterations observed in the present study (Usenko et al. 2007). The lesions identified here are consistent with those reported in fish exposed to environmental contaminants, including pesticides (Sulukan et al. 2017) and heavy metals (Johnson et al. 2007), which have been associated with neurodevelopmental abnormalities, embryonic malformations, and reduced larval viability.

These parallels strengthen the hypothesis that the alterations documented here may be related to chronic exposure to environmental stressors, potentially of anthropogenic origin. Several studies conducted in the Doce River basin have reported high concentrations of metals deposited in the soil and dissolved in river water, including aluminium, iron, and manganese (Oliveira and Camilo 2020; Santana et al. 2026); arsenic, cadmium, lead, and nickel (Gabriel et al. 2021); and copper (Carvalho et al. 2017). Considering that *P. nattereri* and *P. costatus* are non‐native species of substantial nutritional importance, the presence of neural alterations raises broader ecological and public health concerns. Furthermore, Çomakli et al. (2018) demonstrated negative effects in fish exposed to the herbicide glufosinate, including embryonic mortality, delayed hatching, and the induction of brain malformations. Structural disorganization in integrative regions such as the telencephalon and optic tectum is particularly concerning, given their roles in sensory processing, behavioural responses, and environmental adaptation. Therefore, the neural alterations observed in the present study highlight the need for further investigations examining the relationships between environmental contamination, neurodevelopmental abnormalities, and fish population health in the Doce River basin.

## 5 Conclusion

Despite a long history of environmental impacts, stretches of the Doce River and tributaries continue to support reproductive activity in both native and non‐native fish. However, non‐native species exhibited higher frequencies of mature and spawned gonadal maturation stages (SGM), potentially affecting the reproductive balance and long‐term persistence of native populations. Reproductive peaks during the rainy season reflect favourable abiotic conditions and highlight the high reproductive importance of these habitats for regional fish assemblages. Histological analyses revealed gonadal alterations, while morphological and histological evaluations of larvae identified neural abnormalities, including tissue disorganization, altered neuronal architecture, and encephalic tissue eversion. Although the lower Doce River exhibits signs of ecological resilience 10 years after the Fundão Dam disaster, persistent reproductive and developmental impairments suggest that the effects of environmental degradation may continue to influence fish populations and ecosystem functioning. Because similar reproductive and neural alterations were detected in multiple species occupying different ecological niches, these findings support the use of fish assemblages as effective indicators of ecosystem‐level environmental quality in the lower Doce River basin. Continued monitoring of reproductive and developmental biomarkers will be essential for evaluating the long‐term recovery and ecological integrity of this historically impacted watershed.

## Ethics Statement

The research was approved by the Committee on Ethics in the Use of Animals (CEUA) of the University Federal of Viçosa (UFV), under protocol number 36/2019.

## Conflicts of Interest

The authors declare no conflicts of interest.

## Data Availability

Data supporting the results of this study are available upon request to the author for correspondence.
